# Micro solid oxide fuel cell fabricated on porous stainless steel: a new strategy for enhanced thermal cycling ability

**DOI:** 10.1038/srep22443

**Published:** 2016-03-01

**Authors:** Kun Joong Kim, Byung Hyun Park, Sun Jae Kim, Younki Lee, Hongyeul Bae, Gyeong Man Choi

**Affiliations:** 1 Department of Materials Science and Engineering/Fuel Cell Research Center Pohang University of Science and Technology (POSTECH), 77 Cheongamro, Nam-gu, Pohang, Gyeongbuk 37673, Republic of Korea; 2 Research Institute of Industrial Science and Technology (RIST) Pohang 790-330, Republic of Korea

## Abstract

Miniaturized solid oxide fuel cells (micro-SOFCs) are being extensively studied as a promising alternative to Li batteries for next generation portable power. A new micro-SOFC is designed and fabricated which shows enhanced thermal robustness by employing oxide-based thin-film electrode and porous stainless steel (STS) substrate. To deposit gas-tight thin-film electrolyte on STS, nano-porous composite oxide is proposed and applied as a new contact layer on STS. The micro-SOFC fabricated on composite oxide- STS dual layer substrate shows the peak power density of 560 mW cm^−2^ at 550 °C and maintains this power density during rapid thermal cycles. This cell may be suitable for portable electronic device that requires high power-density and fast thermal cycling.

Miniaturized solid-oxide fuel cells (micro-SOFCs), designed using thin-film techniques can attain high specific energy (W h kg^−1^) and energy density (W h L^−1^) and may partially replace Li batteries in portable devices if fabricated successfully^1^. For practical micro-SOFCs, structural design, substrate and materials of membrane electrode assembly (MEA, or cathode/electrolyte/anode) must be carefully considered^1^,^2^. Micro-SOFCs can be of either free-standing membrane (or electrolyte) or supported-membrane type. Free-standing membranes are typically fabricated on Si wafers. An ultra-thin and dense electrolyte membrane is deposited on an Si wafer and electrodes are deposited on the electrolyte membrane after removing the Si by lithography and etching. With well-controlled thin-film MEA of typical geometry, Pt/yttria-stabilized zirconia (YSZ)/Pt cells can achieve power density ≥1 W cm^−2^ at 500 °C^3^,^4^. However, they have extremely short life time, mainly due to instability of nano-porous metal electrodes^3^,^4^,^5^ (Table 1). In addition, free-standing membranes frequently buckle or fracture during fabrication and operation, so the survival probability of the free-standing membrane upon thermal cycling is poor^6^. These problems may arise due mainly to the low thermal expansion coefficient (TEC) of Si substrate (2.5 ppm K^−1^) compared with the thin-film MEA (10–20 ppm K^−1^). Thin-film oxide (Gd-doped CeO_2_) electrode^7^, tapered Si membrane support^8^ or metallic grid support^5^ were introduced to enhance thermomechanical stability of Si-based micro-SOFCs, however thermal cycling ability of free-standing membrane has not been shown to date^1^. More efforts are now being made to increase the stability of cell. In this respect, the supported-membrane design is superior because it can provide the mechanical strength required to support the thin-film membrane. Various porous materials have been used as substrate for micro-SOFCs^9^,^10^,^11^,^12^,^13^,^14^. Anodized aluminum oxide (AAO) is an example of a porous substrate^9^,^10^,^11^,^12^. The main challenges of this type are that a gas-impermeable electrolyte must be deposited on porous AAO substrate, and that they degrade rapidly due to poor thermal stability of nano-structured AAO and metal electrode. Another approach uses currently-available SOFC platforms, e.g., porous cermet anode as a substrate. A thin-film anode, electrolyte and cathode on a conventional Ni-YSZ substrate achieved high power density (588 mW cm^−2^) at 500 °C and good thermal stability (17%/100 h) at 600 °C^13^, but in this cell, the brittle cermet support can crack upon repeating thermal and redox cycles. Fabrication of micro-SOFC on porous metal substrate has been investigated as a way to overcome these problems^15^,^16^. A micro-SOFC supported on porous Ni/stainless steel (STS) dual-layer showed negligible degration of power for 112 h^16^, but thermal cycling ability of the micro-SOFC with this design was not investigated. A nanoporous Ni contact layer with high thermal expansion coefficient (TEC, ~16.5 ppm K^−1^ at 435 °C) is positioned between YSZ (~10.5 ppm K^−1^) and STS (~11.5 ppm K^−1^), so differential expansion and contraction during thermal cyclings may cause fracturing. Here, we use an alternative contact layer, La-doped SrTiO_3_ (LST), due to its TEC comparable to that of the YSZ and STS, high electronic conductivity and redox stability. If this material is mixed with YSZ, both good compatibility and nanoporous microstructure can be obtained due to limited sintering or grain growth. Thus LST-YSZ composite may be appropriate as a substrate to deposit gas-impermeable thin-film electrolyte. Furthermore, the exsolution technique was used to utilize composition of (La, Sr)(Ti, Ni)O_3_ (LSTN) to improve electrical conductivity and catalytic activity^17^,^18^. Finally, a newly designed dual-layer substrate (LSTN-YSZ/STS) was fabricated by simple co-firing. Then a thin-film of oxide-based electrode and electrolyte was deposited in a manner similar to one described previously^16^. To the best of our knowledge, this is the first demonstration of the ability of the thermal robustness of micro-SOFC, which has never been attained in many conventional Si-based devices.

## Experimental procedures

**Figure 1:**
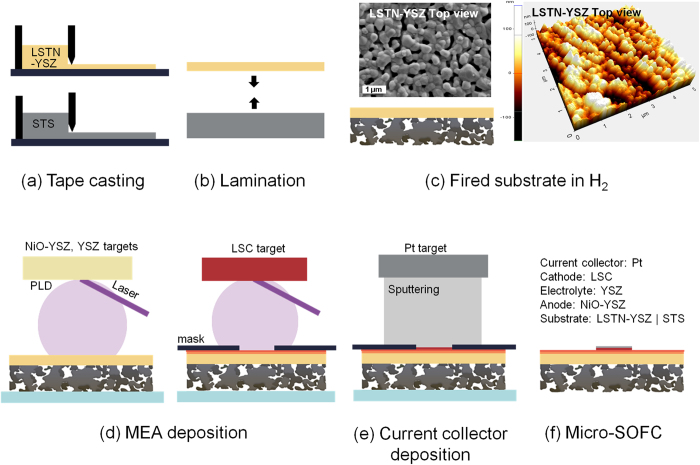
Schematics of fabrication process of micro-SOFC supported on LSTN-YSZ/STS substrate.

The fabrication process is simpler than for other micro-SOFCs because it does not use complicated lithography, etching or templating (Fig. 1, Table 1). A dual-layer substrate is prepared using conventional tape-casting (Fig. 1a) and lamination (Fig. 1b). Then the green dual-layer is co-fired in reducing gas (Fig. 1c) to avoid oxidation of STS. To ensure suitable nanostructure, the dual-layer substrate is characterized after firing. The LSTN-YSZ contact layer (thickness ~40 μm) has pore size of ~500 nm and surface root-mean-square roughness (RMS) of 44 nm (Fig. 1c). An area porosity *ε* of LSTN-YSZ surface obtained from binary images, increased from 14 to 18% after surface polishing and a RMS value decreased from ~44 nm to ~21 nm after surface polishing (Supporting Information, Fig. S1), thus the porosity and surface roughness are appropriate for deposition of 2-μm-thick and dense electrolyte.

X-ray diffraction confirmed that LSTN-YSZ on STS after firing exhibited all major peaks of single-phase SrTiO_3_ and cubic YSZ (Supporting Information, Fig. S2). A 380-μm-thick STS support has 50-μm particles that are connected to each other well enough to provide mechanical support for the micro-SOFC (Supporting Information, Fig. S3). The pore size ~10–100 μm and *ε* ~28% are enough to let fuel gas flow in and reaction by-products (H_2_O) flow out. Further characterization of the dual-layer substrate includes its conductivity as a function of temperature, its area-specific resistance (ASR), and its stability during 100 h at operation temperature and under thermal cycling. The electrical conductivity of the dual-layer substrate was measured with temperature (400–550 °C) in wet H_2_ (97% H_2_ + 3% H_2_O) (Supporting Information, Fig. S4). Due to its metallic conductivity, we assumed that STS does not contribute to the substrate resistance. The conductivity of the LSTN-YSZ increased with temperature (*E*_a_ ~ 0.39 eV) and was two to three orders of magnitude higher than that of YSZ^19^ and one order of magnitude lower than that of LSTN^20^. The conductivity of the LSTN-YSZ composite mainly due to that of the high proportion of LSTN (70 wt%) and its percolation though the porous composite. The LSTN-YSZ had ASR ~0.02 Ω cm^2^ after short-term operation (550 °C, 100 h) and fast thermal cycling (40 °C min^−1^, 150–550 °C); this ASR is smaller than the usual target value of Ohmic ASR (~0.15 Ω cm^2^). The MEA was deposited by pulsed laser deposition (PLD), with NiO-YSZ as the anode, YSZ as the electrolyte and La_0.7_Sr_0.3_CoO_3-δ_ (LSC) as the cathode (Fig. 1d), then a current collector (Pt) was sputter deposited (Fig. 1e) to result in a micro-SOFC (Fig. 1f). Further details in experimental procedure can be found in Supporting Information.

## Results and Discussion

**Figure 2:**
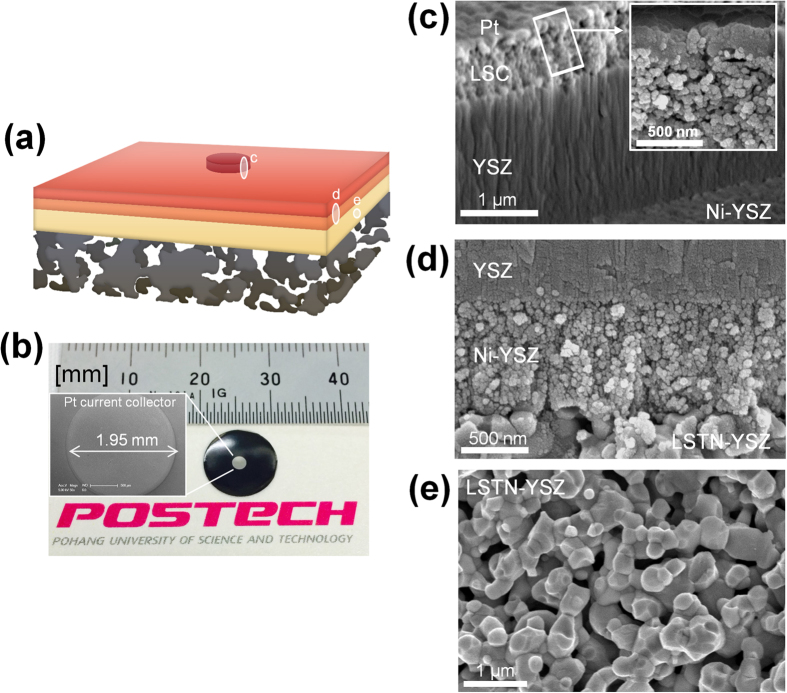
Image of micro-SOFC. (**a**) Schematic of thin-film MEA supported on porous STS substrate. (**b**) Photograph of cell. A logo is a trademark of Pohang University of Science and Technology (POSTECH) and is protected by copyright; it is used in this figure with permission. Cross-sectional S.E.M. image of (**c**) Pt/LSC/YSZ (Inset: magnified view of Pt/LSC) (**d**) YSZ/Ni-YSZ/LSTN-YSZ, (**e**) LSTN-YSZ contact layer. (**c–e**) also correspond to (**c–e**) in Fig. 2a.

Total area of a cell (Fig. 2a,b) was 78 mm^2^, and the cathode area was 3 mm^2^. The active area could be enlarged by careful control of surface defects in the LSTN-YSZ contact layer. Due to the robust STS support, the cell provides good mechanical stability, ease of handling, and flexibility. Thin Ni or STS-supported cells are mechanically flexible without visible cracks on the electrolyte^21^; therefore, this type of cell may show mechanical stability when stacked vertically, thereby overcoming one of the shortcomings of conventional SOFCs. The microstructure of a cell was observed after electrochemical tests. A thin-film MEA consists of 0.7-μm-thick LSC, 2-μm-thick YSZ (Fig. 2c) and 0.6-μm-thick Ni-YSZ (Fig. 2d). The YSZ electrolyte looks dense and has no pinholes. Both electrodes have similar nanostructured grain size or pore size (<100 nm). The rough surface of STS was covered by LSTN-YSZ, which has small and uniform pore size (<0.5 μm, Figs 2e and 1c).

Pore size and surface smoothness of a substrate significantly affect the structural stability and morphology of thin films^22^. In a preliminary study, we used Ni-YSZ as a nano-porous contact layer instead of LSTN-YSZ as similarly shown in the literature^23^,^24^. However, the pore size and porosity of the Ni-YSZ are ~1 μm and 27%, respectively, and both are higher than those of LSTN-YSZ. Because Ni particles are easily sintered during co-firing in a reducing atmosphere^25^; the resulting surface was not appropriate as a target for deposition of 2-μm-thick MEA. Cr poisoning during cell firing mostly degrades Ni-YSZ anode and thus it is not quite significant in this study since we have deposited Ni-YSZ on pre-fired substrate. However we have analyzed the mutual elemental diffusion between LSTN-YSZ and STS of as-fired (1250 °C) bi-layer substrate. There was negligible Fe or Cr diffusion from STS layer into LSTN-YSZ layer. Thus LSTN-YSZ can be used as diffusion barrier layer (DBL). We have previously confirmed that YST (Y-doped SrTiO_3_)-CeO_2_ layer can be used as DBL^25^. Thus, we confirm that a LSTN-YSZ/STS dual-layer fabricated by simple co-firing is a suitable porous substrate to meet microstructural requirements, electrical properties, thermal stability, and chemical stability.

**Figure 3:**
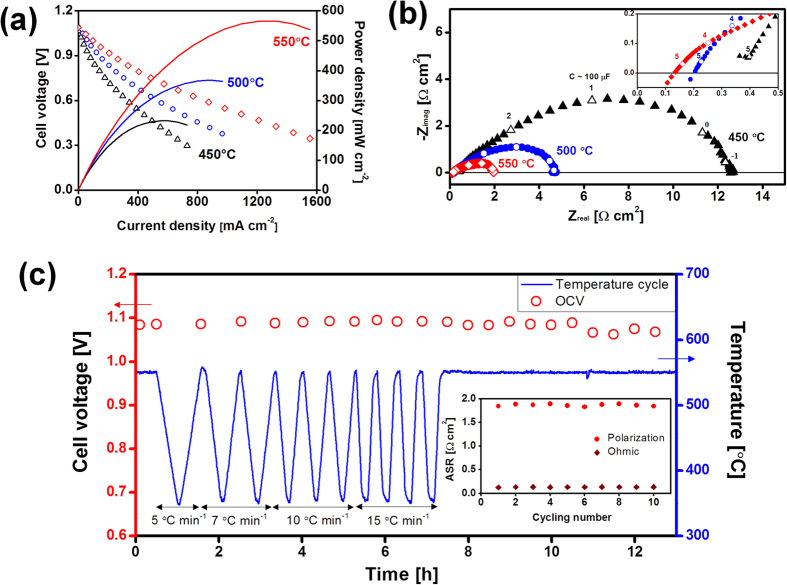
Electrochemical performance and thermal cycling stability of the micro-SOFC. Wet H_2_ gas (97% H_2_ + 3% H_2_O mixture) was supplied as fuel gas to the anode (60 cm^3^ min^−1^) and open air was used as oxidant gas. (**a**) I (current)-V (voltage) and I-P (power density) curves **(b)** Impedance spectra at 450, 500, and 550 °C. Inset: detailed view in the high-frequency range to show Ohmic resistance. Numbers on the curve: log (frequency [Hz]). (**c**) Thermal cycling test between 350 and 550 °C with 5–15 °C min^−1^ heating and cooling rates. Electrochemical measurements (OCV and impedance) were conducted after every thermal cycle. OCV (circles) and area specific resistance (ASR) were maintained during 10 thermal cycles and after cell operation for 6 h.

Current-voltage (*I*-*V*) curves of the cell at 450–550 °C show that the open-circuit voltage (OCV) was >1.05 V (Fig. 3a, left y axis). This high voltage indicates that the thin-film electrolyte is quite dense without pinholes or cracks, and has a good seal. For micro-SOFCs fabricated on porous substrate, a reasonably high OCV value (≈Nernst voltage) is often limited due to large pore size, roughness, and defects of the deposition surface or to electrolyte damage during lithography and etching^26^. However, the substrate used in this study is fabricated without lithography and etching, and the YSZ/Ni-YSZ films are thick enough (≥2 μm) to close the pore openings (~500 nm) of the LSTN-YSZ substrate (Fig. 2e). To the best of our knowledge, only one study reported OCV >1 V with 3–5 μm thick YSZ electrolyte which was sputtered on Ni-YSZ/STS substrate^23^. The peak power density (PPD) was 235, 370 and 560 mW cm^−2^ at 450, 500 and 550 °C (Fig. 3a, right y axis).

The contributions of Ohmic ASR *ASR*_Ω_ and polarization ASR *ASR*_p_, were extracted from the impedance spectra (IS) of the cell measured under open-circuit condition (Fig. 3b). The high-frequency intercept (Fig. 3b, inset) and low-frequency intercept are associated with *ASR*_Ω_ and *ASR*_P_, respectively. The *ASR*_Ω_ value was used to calculate that the ionic conductivity of the 2-μm-thick YSZ film was 9.74 × 10^−4^ S cm^−1^ at 500 °C, which is comparable to that of bulk YSZ (1.1 × 10^−3^ S cm^−1^) at 500 °C^19^. The IS patterns show that the total ASR *ASR*_tot_ of the cell is primarily determined by *ASR*_P_, which was 97, 96 and 93% of *ASR*_tot_ at 450, 500, and 550 °C, respectively; this high percentage confirms that the resistance of the cell is limited by *ASR*_P_. Although further interpretation of limiting factor of reactions pathways for thin-film electrode was not possible at the present study, microstructure and thickness of the films may have important contributions. For example, deposition of same thin-film compoment on nano-porous Ni substrate with 6-μm-thick Ni-YSZ, 2-μm-thick YSZ and 6-μm-thick LSC achieved PPD of 110 mW cm^−2^ at 570 °C^14^. Although indirect, this comparison indicates that microstructure and thickness of thin-film electrode for the present cell may have more reaction sites (TPB) and sufficient gas transport than aforementioned cell.

The thermal stability of the cell was tested by applying ten thermal cycles between 350–550 °C up to 15 °C min^−1^ durations at 550 °C for 6 h (Fig. 3c). To check thermal robustness of the cell during the test, OCV was monitored *in-situ* during the entire test time, and IS and Current-voltage-power (*I*-*V-P*) curves were measured between each pair of thermal cycles at 550 °C. During the first ten thermal cycles, OCV was >1 V; this consistency indicates that cracks were not generated in the YSZ electrolyte. The state-of-the-art free-standing micro-SOFC has a huge thermal mismatch between free-standing electrolyte and Si substrate, so thermal cycling may damage the free-standing memebrane. Nonetheless, in a recent study, rapid thermal cycling ability of Si-supported cells caused no membrane fracture over several cycles, but specific changes in OCV were not reported so the stability of the YSZ electrolyte has not been established^7^. In contrast, we confirmed negligible OCV degradation under rapid thermal cycles. ASR change was also observed during thermal cycling (Fig. 3c, inset); little degradation was observed in either *ASR*_Ω_ or *ASR*_P_. Therefore, an initial peak power density was remained at the end of the thermal cycling. The stable *ASR*_Ω_ means that the cell has a stable interface without electrolyte cracks or de-lamination between the cell components, and also has little interfacial reaction. The stable *ASR*_P_ might be utilized to eliminate time-dependent microstructure degradation of thin-film electrode, i.e., densification of nano-porous Pt electrodes, which is a major problem in typical micro-SOFC devices (Pt/YSZ/Pt on Si)^3^,^4^,^5^. Because we used an oxide-based electrode, the densification of thin-film microstructure was limited. A Ni-YSZ thin-film anode requires post-annealing at high temperature (1200 °C) to stabilize its microstructure, because the Ni-YSZ thin-film has a large driving force for Ni coarsening caused by minute equiaxed crystallites (diameter of several nanometers)^27^. However, the Ni-YSZ film used in this study showed little Ni coarsening without annealing at high temperature and operation at 550 °C for 13 h (Fig. 2d); this novel finding will be discussed in future work. For LSC thin-film cathodes, chemical compatibility with YSZ at such a low temperature could be a concern because Noh *et al.* suggests necessity of GDC buffer layer between Co-containing cathode, e.g. LSC, and YSZ due to the formation of insulating phase even at the temperature less than 650 °C^28^. We are currently fabricating the cell with GDC buffer layer and the results will be reported.

Thermal stability of multi-layered devices such as SOFCs is determined primarily by thermal stress between cell components, as a consequence of mismatched TECs. However, for small cells (area ~78 mm^2^) and thin layers (thickness ≤50 μm, excluding STS layer) like the present cell, the cell is resistant to failure caused by cracking or delamination due to the temperature gradient. In contrast, a conventional anode-supported cell (ASC), i.e. Ni-YSZ supported cell, often cracks after cooling to room temperature although the cell size is small. The cracks occur due to TEC mismatch between YSZ electrolyte and the sealants or cell holder (alumina tube). The TEC of the commercially-available SOFC sealant used in this study is 12.6 ppm K^−1^, which is reasonably-matched with that of the main cell structure: YSZ (10.5 ppm K^−1^), Ni-YSZ (12.5 ppm K^−1^), LSTN-based layer (11–12 ppm K^−1^) and STS (11.2 ppm K^−1^)^29^,^30^. However, the alumina tube has much lower TEC (7.9 ppm K^−1^) than the cell components; the mismatches in TEC may be the cause of cracking during thermal cycle. A 50 μm-thick YSZ electrolyte layer (9-mm diameter), with additional 30 and 40 μm-thick cathode and anode, respectively, supported on a 2 mm-thick YSZ ring by using YSZ paste survived without cracking and thus no severe OCV and ASR degradation after thermal cycles between 200–800 °C (50 °C min^−1^)^31^; the authors predicted that 1-cm-thick YSZ with YSZ electrolyte—YSZ ring—YSZ paste configuration can survive up to 200 °C min^−1^ thermal cycles. Thus cracks are induced mostly due to TEC mismatch between YSZ and cell components or sealants. Surprisingly, the current cells never crack during fast thermal cycles or even cooling to room temperature. We speculate that the porous but ductile STS substrate may absorbed the thermomechanical stress caused by sealants or the alumina tube during thermal cycles. This resistance to cracking demonstrates the thermal robustness of micro-SOFC supported by porous STS. Among the cell components, the LSC cathode has the highest TEC of 21.3 ppm K^−1^, so de-lamination of the LSC film from an electrolyte surface may be expected. Nonetheless, due to the small area of the cathode (3 mm^2^), the ASR of the cell was maintained during thermal cycles.

Among numerous types of micro-SOFCs, successful demonstration of thermal cycling ability has been rare. One paper reported a thermal cycling experiment with a micro-SOFC built on conventional Ni-YSZ substrate; the result was quite encouraging^13^. But this cell may also fail during cooling to room temperature due to the brittle nature of Ni-YSZ cermet as a substrate, and its contact with sealants. We conducted additional durability test and its result is presented in Supporting Information, Fig. S5. Fifty thermal cycles were repeated with wide temperature range (150–500 °C) and heating/cooling rates of 20 °C min^−1^. OCVs were measured *in-situ* during thermal cycles. OCV >1 V was maintained throughout the thermal cycles; this consistency indicates that the electrolyte did not crack. The results again confirm the thermal cycling stability of the present micro-SOFC. Although the tests cannot guarantee the durability of current cell as practical micro-SOFCs, to the best of our knowledge, this is the first demonstration of thermally robust micro-SOFCs fabricated on porous STS substrate.

**Figure 4:**
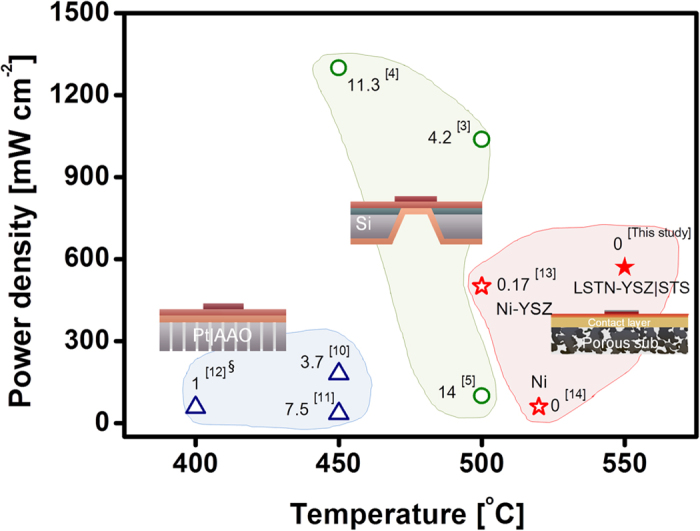
Comparison of literature data in terms of peak power densities (mW cm^−2^) and degradation rate (% h^−1^) of power density at the operation temperature of micro-SOFCs that use YSZ electrolyte. Three different types of cell are categorized with respect to electrode and substrate materials. e.g. (*i)* free-standing Pt/YSZ/Pt on Si^3^,^4^,^5^, (*ii)* Pt/YSZ/Pt on AAO^10^,^11^,^12^ and (*iii)* LSC/YSZ/Ni-YSZ on porous substrate^13^,^14^. Numbers indicate degradation rate (% h^−1^). ^§^Power density and degradation at 0.8 V. For group (*iii*), substrate materials are shown; i.e., Ni-YSZ, Ni, LSTN-YSZ/STS.

A final goal of this research is to fabricate micro-SOFCs that are both durable and produce high power density. In micro-SOFCs that use YSZ electrolyte, the relationship between initial peak power density and operation temperature differs among designs (Fig. 4). The degradation rate [% h^−1^] of peak power density was calculated based on the cell test time (Table 1); values range from 4.2–14% h^−1^ for free-standing Pt/YSZ/Pt on Si^3^,^4^,^5^, 1.0–7.5% h^−1^ for Pt/YSZ/Pt on AAO^10^,^11^,^12^ and 0–0.17% h^−1^ for LSC/YSZ/Ni-YSZ on Ni-YSZ, Ni and LSTN-YSZ/STS substrates^13^,^14^. A Pt/YSZ/Pt cell supported on Si can achieve high power density at low temperature, but rapid degradation is difficult to avoid, mostly due to instability of the nano-porous Pt electrode. A cell supported on AAO has additional instability due to poor thermal stability of AAO. Although use of durable ceramic electrodes in free-standing YSZ membranes has been studied, successful demonstration of both thermal cycling ability and durability has been limited^7^. In contrast, the cell with oxide-based electrode supported on porous substrate in this study shows small degradation rate. The power density of this cell can be further improved by using alternative compositions of thin-film electrode and electrolyte, and by tailoring its microstructure. The realization of micro-SOFC fabricated on porous STS substrate reinforces the feasibility of this technology and may provide a new implementation strategy. Long-term tests with a constant electrical load will be conducted for chemical stability, and fuel versatility will be assessed as ultimate studies of the reliability of micro-SOFCs. The slow and expensive PLD process will also be replaced by sputtering process which allows faster deposition with larger area. Large-size membranes are always favorable when assembling cells to form stack^1^,^32^.

In summary, the fabrication and thermal robustness of a micro-solid oxide fuel cell (micro-SOFC) was demonstrated. An oxide-based thin-film membrane electrode assembly was deposited on top of a dual-layer substrate. The substrate consists of porous LSTN-YSZ as a contact layer to deposit gas-tight YSZ thin-film electrolyte on it and STS as a thermo-mechanical support. The cell attained peak power density of 560 mW cm^−2^ at 550 °C with wet H_2_ fuel gas and maintained this power density during rapid thermal cycling. This cell may be suitable as a power source for small portable electronic devices that require high power density and fast thermal cycling. The results may help to further advance process science and technology of micro-SOFCs that use thin-film components.

## Additional Information

**How to cite this article**: Kim, K. J. *et al.* Micro solid oxide fuel cell fabricated on porous stainless steel: a new strategy for enhanced thermal cycling ability. *Sci. Rep.* **6**, 22443; doi: 10.1038/srep22443 (2016).

## Supplementary Material

Supplementary Information

## Figures and Tables

**Table 1:** Summary and comparison of the fabrication, OCV, power density and degradation of various micro-SOFCs.

Group	Substrate (process./thickness)	Anode (process./thickness)	Electrolyte (process/.thickness)	Cathode (process/thickness)	Active area [mm^2^]	OCV [V]	Power density [mW cm^−2^]	Temp [^o^C]	Degradation rate
Harvard Univ. (Kerman *et al.*)^3^	Si wafer (L&E/−)	Pt (SP/80 nm)	YSZ (SP/100 nm)	Pt (SP/80 nm)	0.03	0.97	1037	500	50%/12 h (at 400 °C)
(Tsuchiya *et al.*)^5^	Si wafer (L&E/−)	Pt (SP/30 nm)	YSZ (SP/54 nm)	LSCF (SP/47 nm)	25 (w/Ni grid)	0.75	155	510	14%/1 h (at 500 °C)
Stanford Univ. (An *et al.*)^4^	Si wafer (L&E/-)	Pt (SP/80 nm)	YSZ|YDC (ALD/60 nm)	Pt (SP/80 nm)	0.002 (corrugated membrane)	1.05	1300	450	34%/3 h (at 400 °C)
Seoul National Univ. (Ha *et al.*)^10^	AAO (−/100 μm)	Pt (SP/ ≤ 380 nm)	YSZ (ALD & SP/390 nm)	Pt (SP/200 nm)	4	1.1	180	450	11%/3 h (at 450 °C)
(Ji *et al.*)^11^	AAO (−/100 μm)	Pt (SP/250 nm)	YSZ|GDC (ALD & SP/460 nm)	Pt (SP/200 nm)	1	1.07	35	450	30%/4 h (at 450 °C)
K.I.S.T. (Kwon *et al.*)^12^	AAO (−/600 nm)	Pt (SP/80 nm)	YSZ|Al_2_O_3_|YSZ (ALD&PLD/900 nm)	Pt (SP/80 nm)	0.01	1.0	90 (at 0.8 V)	400	17%/17 h (at 400 °C)
(Noh *et al.*)^13^	Ni-YSZ (CM, sP/1 mm)	Ni-YSZ (PLD/2–3 μm)	YSZ|GDC (PLD/600 nm)	LSC-GDC/LSC (PLD/5 μm)	100	1.1	588	500	17%/100 h (at 600 °C)
Houston Univ. (Chen *et al.*)^14^	Ni foil (L&E/6 μm)	Ni-YSZ (PLD/6 μm)	YSZ (PLD/2 μm)	LSC (PLD/6 μm)	–	0.8	110	570	0%/6 h (at 520 °C)
This study	LSTN-YSZ (40 μm)/STS 434 L 380 μm (TC)	Ni-YSZ (PLD/600 nm)	YSZ (PLD/2 μm)	LSC (PLD/700 nm)	3	1.0	560	550	0%/13 h (at 550 °C)

Notation: PM: powder metallurgic process, CM: compression-molded, sP: screen printing, SP: sputtering, TC: tape casting, PLD: pulsed laser deposition, ALD: atomic layer deposition, L&E: lithography and etching.
