# Supplementary material for: Micro solid oxide fuel cell fabricated on porous stainless steel: a new strategy for enhanced thermal cycling ability
Source: Sci Rep. 2016 Mar 1;6:22443. doi: 10.1038/srep22443 (PMC4772004; doi:10.1038/srep22443)
Supplement: Supplementary Information [file srep22443-s1.doc]

Supporting information

Micro solid oxide fuel cell fabricated on porous stainless steel: a new strategy for enhanced thermal cycling ability

Kun Joong Kim, Byung Hyun Park, Sun Jae Kim, Younki Lee, Hongyeul Bae and Gyeong Man Choi*


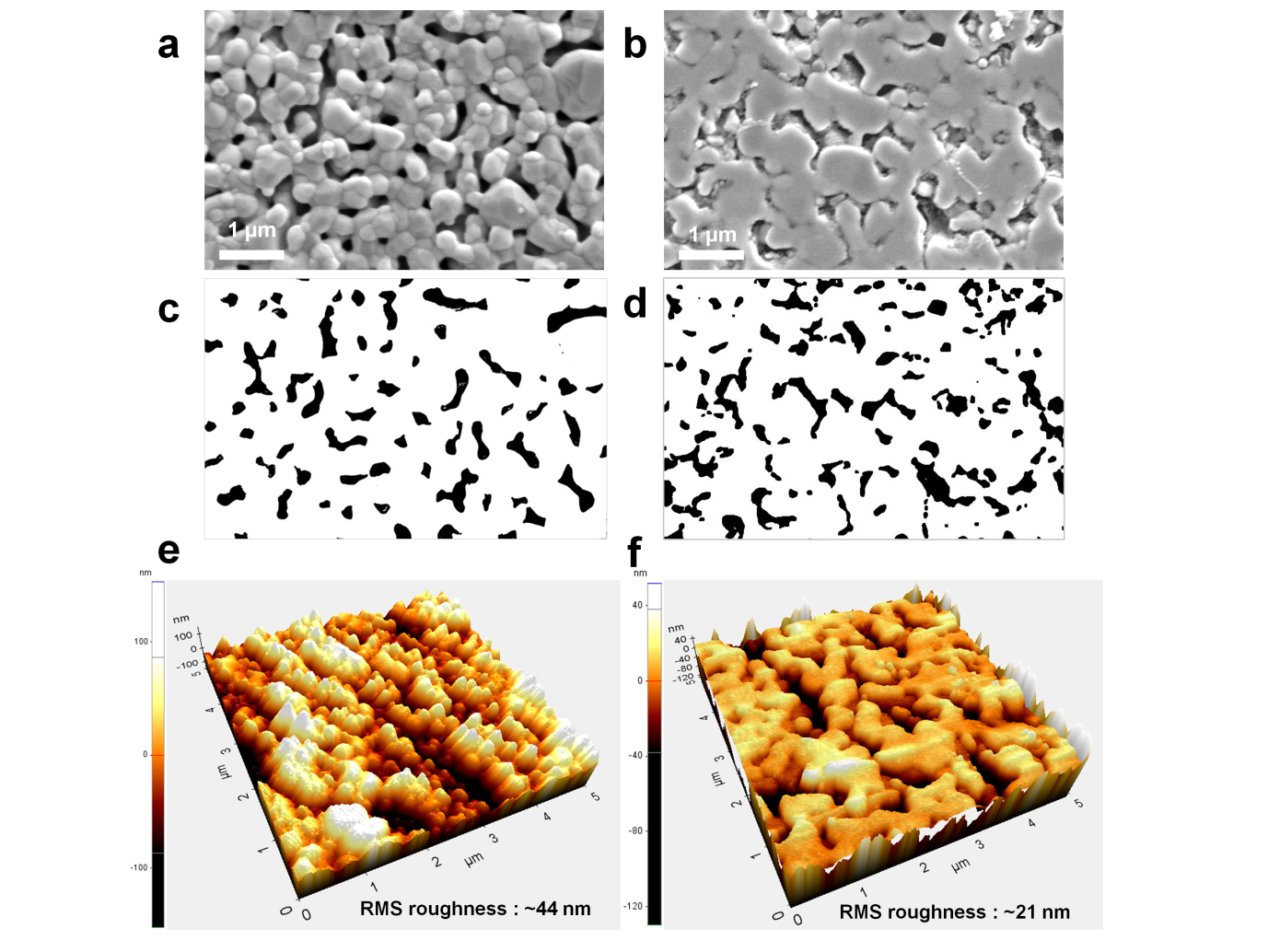


**Figure S1.** SEM micrograph (a, b), corresponding binary converted image (c, d), and AFM micrograph (e, f) of LSTN-YSZ top surface; after co-firing (a, c, e) and after polishing (b, d, f).


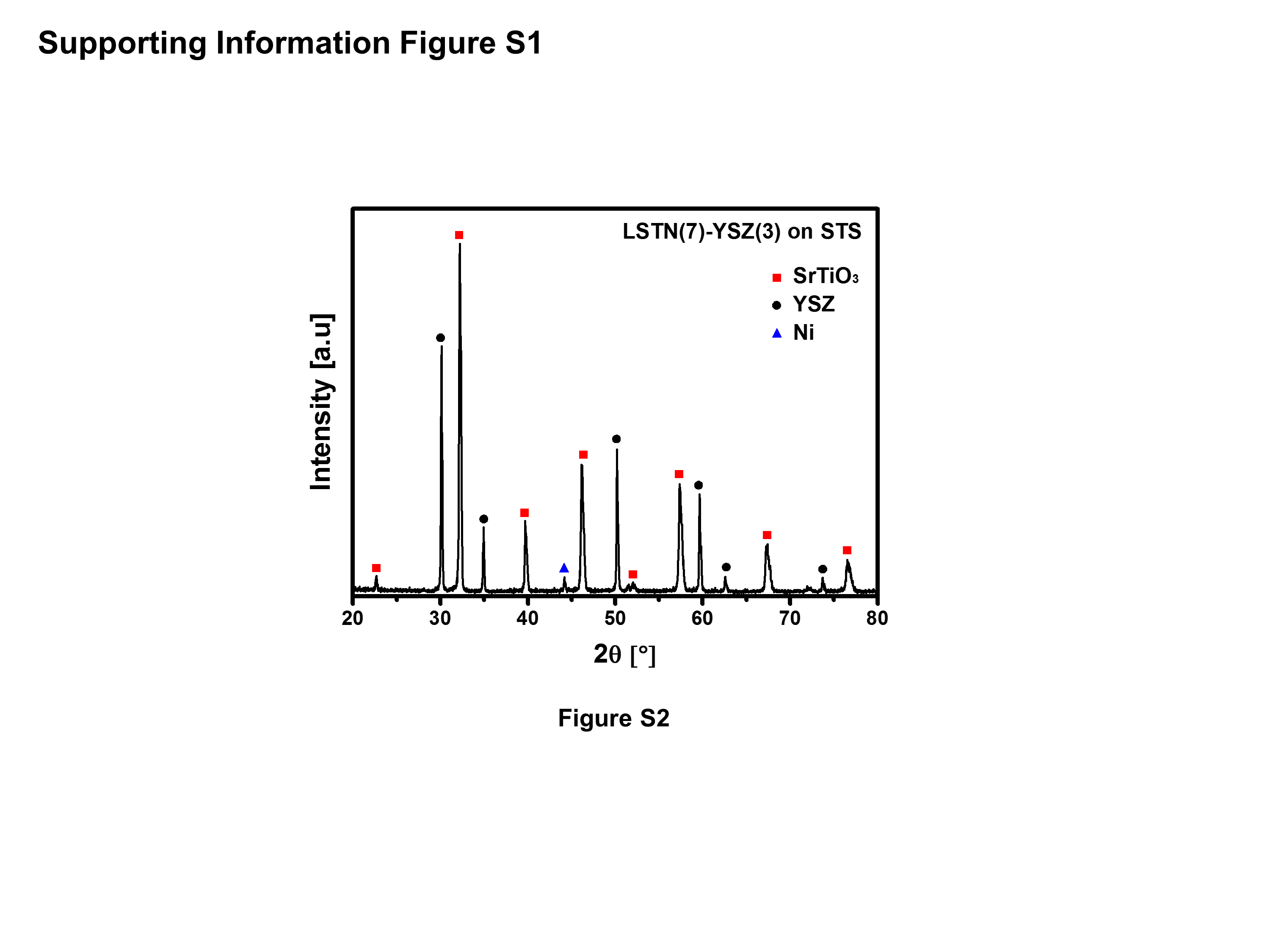


Figure S2. X-ray diffraction (XRD) patterns of porous LSTN-YSZ contact layer (70 : 30 wt. %) coated on STS layer after co-firing at 1250 °C in dry H_2_.


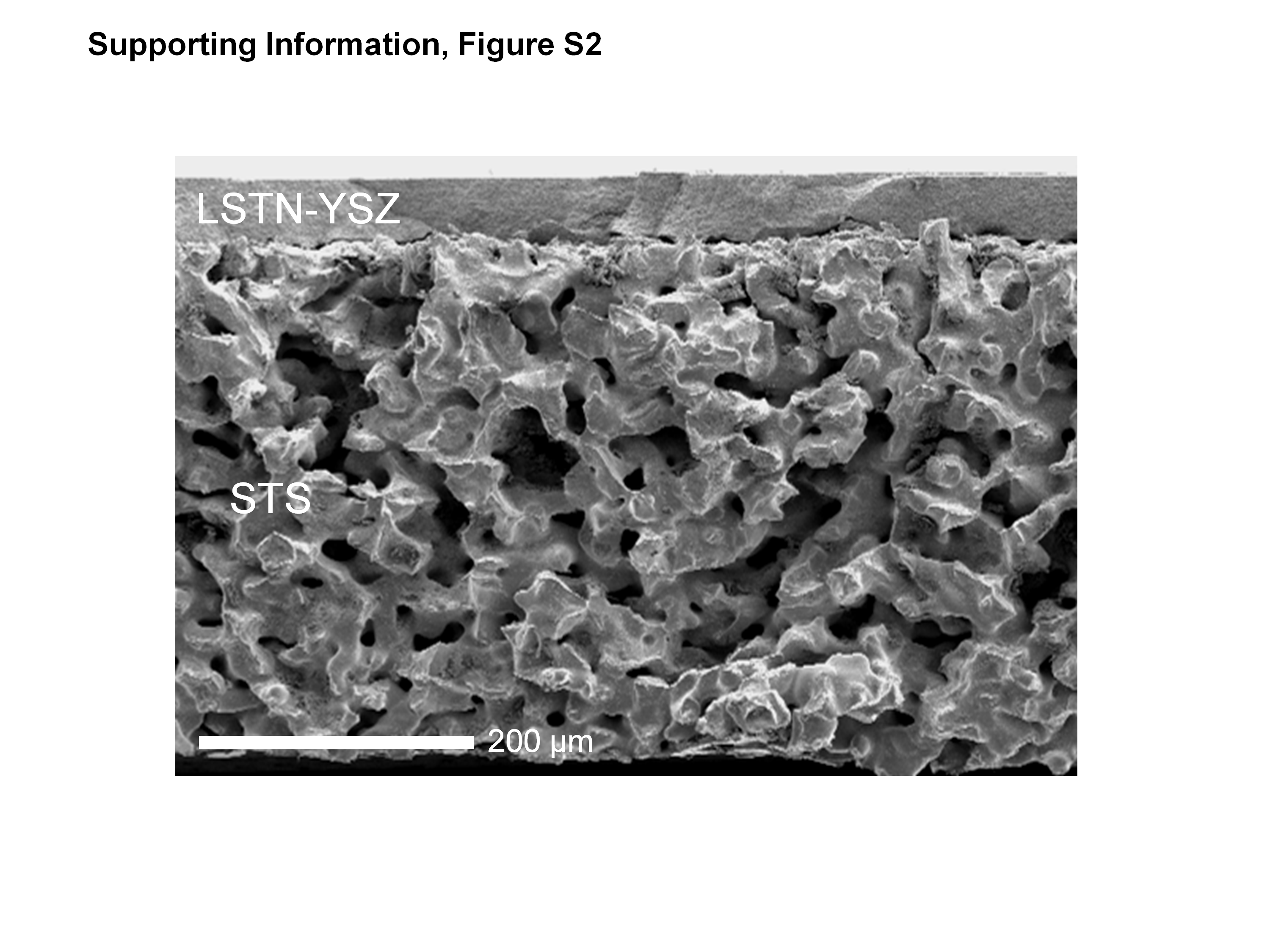


Figure S3. Cross-sectional S.E.M. image of porous LSTN-YSZ/ STS substrate.


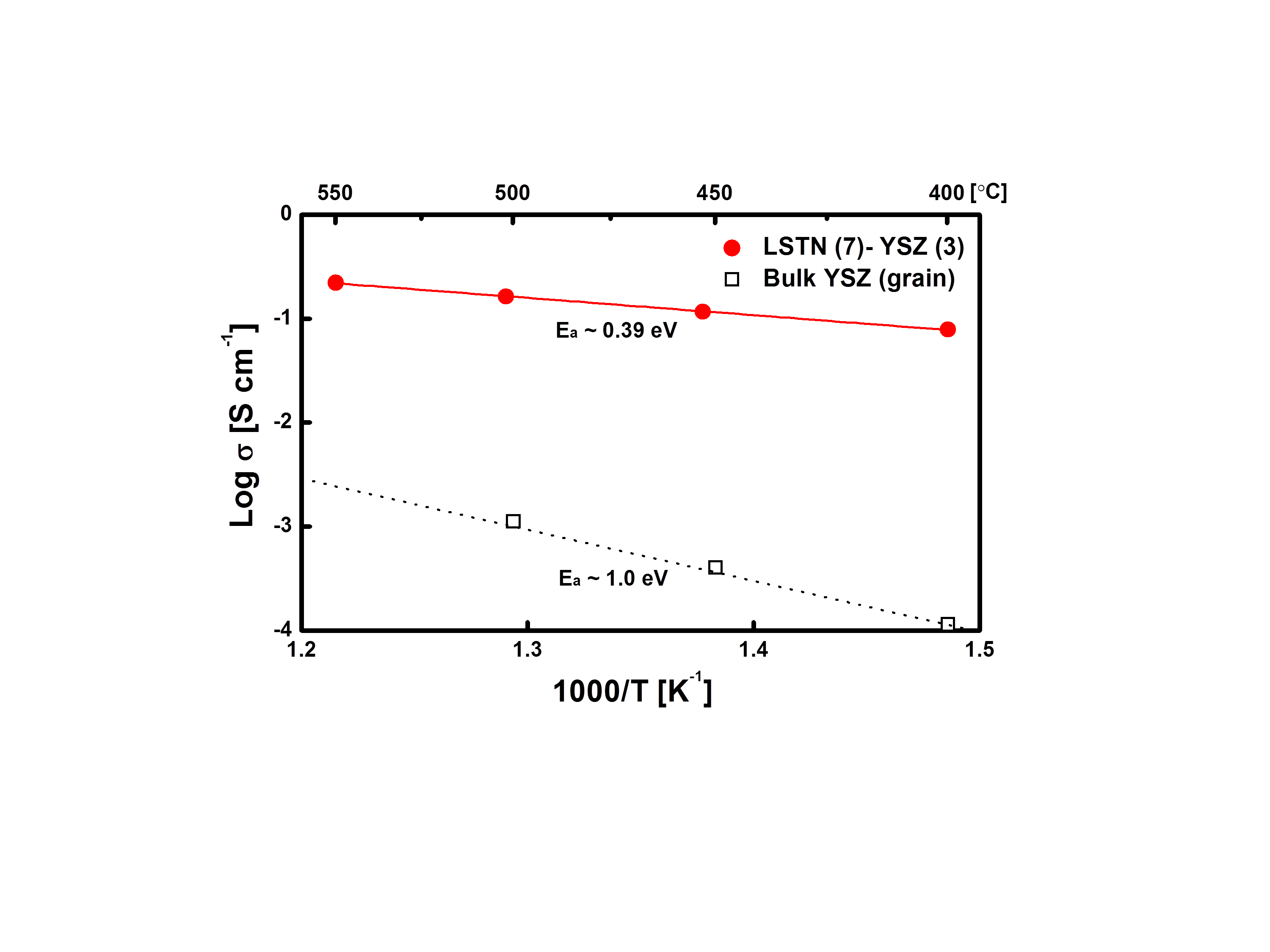


Figure S4. Electrical conductivity versus inverse of temperature (400-550 °C) for porous substrates (LSTN-YSZ/ STS) measured in wet H_2_ (97% H_2_ + 3 % H_2_O), sintering condition: 1250 °C, dry H_2_. Conductivity of bulk YSZ (grain) sintered in air and measured in air is shown for comparison.


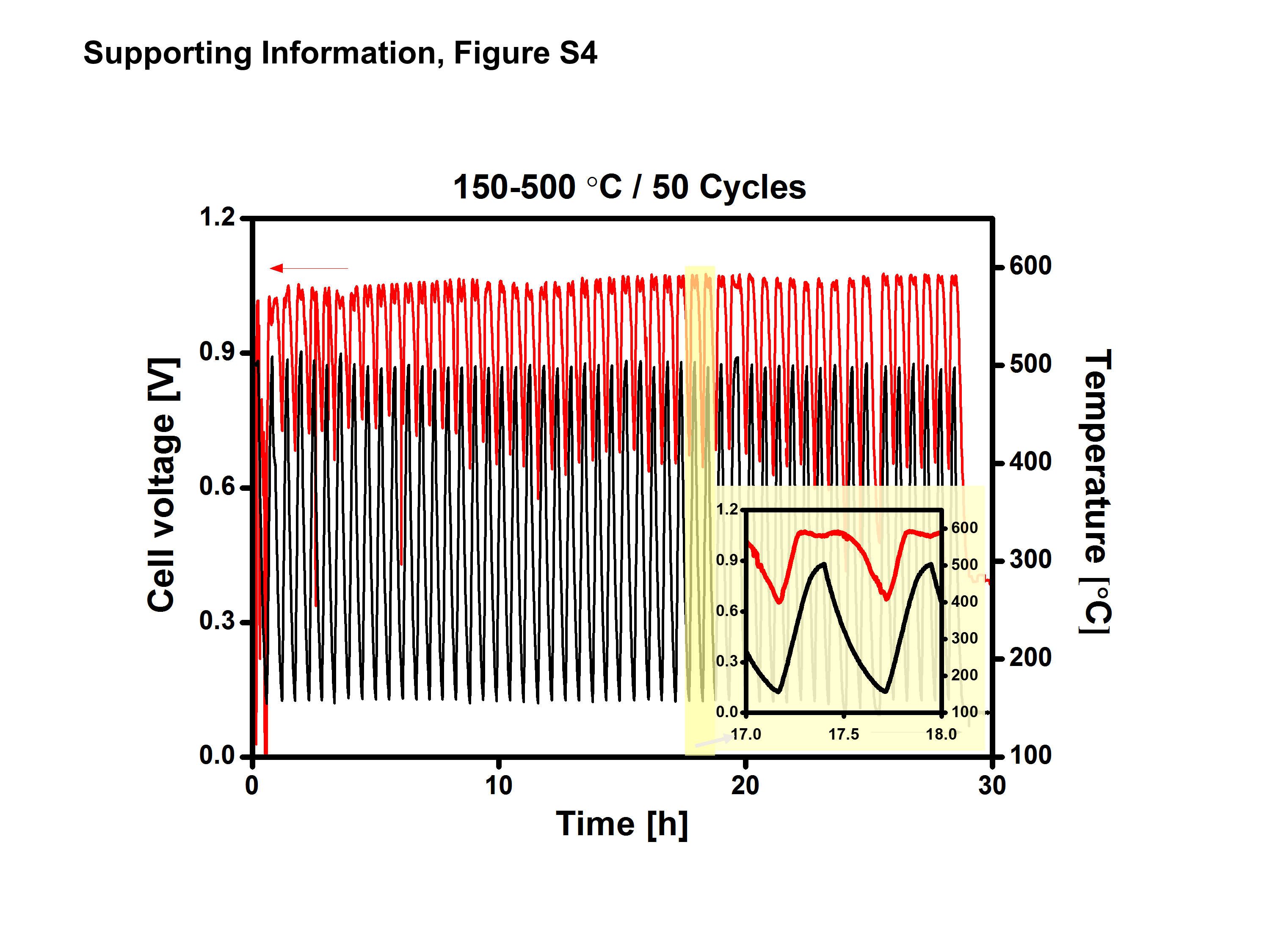


Figure S5. Cell voltage during 50 thermal cycles. Wet H_2_ gas (97 % H_2_ + 3 % H_2_O mixture) was supplied as fuel gas to the anode (60 cm^3^ min^-1^) and open air was used as oxidant gas. Each thermal cycle is between 150 and 500 °C with heating and cooling rates of ~20 °C min^-1^. OCV measurements were conducted in-situ during 50 thermal cycles. OCV (red line) ~1 V was maintained throughout thermal cycles; i.e., the electrolyte did not crack. Inset: magnified view of two thermal cycles for 1h. The required time for one cycle (heating and cooling) is ~30 min. Cycling was stopped after 50 cycles because no further change was observed.

Experimental details

*Materials preparation*

Commercial STS powder (AISI 434L, 400-500 mesh) [Fe/ 16.5Cr/ 1Mo/ 0.2Ni/ 0.1Mn/ 0.005S/ 0.017C/ 0.025P/ 0.8Si] (Höganäs AB, Sweden) was used for fabrication of the metal support. La_0.2_Sr_0.8_Ti_0.9_Ni_0.1_O_3-δ_ (LSTN) powder was synthesized using solid-state reaction(^16^ in main text). The LSTN and Y_2_O_3_-stabilized ZrO_2_ (YSZ, 0.3 μm, TZ-8YS, Tosoh, Japan) powder were planetary-milled (Pulverisette 6, Netzsch, Germany) in 70: 30 wt. % ratio to form a composite. To prepare oxide targets of thin-film anode for PLD, NiO (1 μm, 99.97 %, Kojundo chemical, Japan) and YSZ powder were planetary-milled in 60: 40 wt. % ratio, die-pressed to form a pellet, then sintered at 1400 °C for 3 h in air. Target preparation for YSZ and La_0.7_Sr_0.3_CoO_3-δ_ (LSC) can be found elsewhere (^15^ in main text).

*Device fabrication*

Button-type micro-SOFCs were fabricated using a combination of thick- and thin-film processes. Tape casting was used to fabricate the thick-film support that consisted of dual-layer of ~40-μm-thick LSTN-YSZ and ~380‑μm-thick STS. To prepare the slurry for tape casting, LSTN-YSZ composite powder and STS powder were mixed with binder solution composed of toluene and ethanol as solvents, polyvinyl butyral (PVB, B-76) as a binder (10 wt. % of raw powders), and dioctyl phthalate (DOP) as a plasticizer (50 wt.% of binder contents). Corn starch was added to the STS paste as a pore-forming agent. The mixture of powders and binder solution was milled with zirconia balls (diameters 10 mm and 5 mm) for 72 h. Green sheets of LSTN-YSZ and STS were cast as 30-40-μm-thick tapes after drying. The green sheets were laminated at 30 MPa and 60 °C for 20 min to achieve the desired thickness, then punched out to yield circular green-cells of 11-mm diameter. Binders in tapes were burned out at < 400 °C for 12 h and the green cells were co-fired at 1250 °C for 3 h in dry H_2_ atmosphere. To prevent deformation of the sample during sintering, a porous ZrO_2_ plate was used to apply a vertical load (1.5 g cm^-2^) to the green cell. To remove surface defects, the surface of LSTN-YSZ was polished using SiC paper and diamond suspension down to 0.25 μm. On top of the porous LSTN-YSZ/ STS support, thin film MEA was deposited using pulsed laser deposition (PLD); the MEA includes Ni-YSZ as an anode, YSZ as an electrolyte and LSC as a cathode. A KrF-excimer laser source (wavelength 248 nm, energy density 1.9 J cm^-2^, pulse repetition rate 10 Hz) was used to ablate the prepared target for the film deposition. The base pressure in the vacuum chamber was 8 × 10^-4^ Pa. The distance between target and substrate was ~55 mm. The NiO-YSZ and YSZ film were sequentially deposited onto porous LSTN-YSZ surface at substrate temperature T_sub_ = 600 °C and oxygen pressure P = 4-6.7 Pa. Then the sample was cooled to room temperature at 3 °C min^-1^. The LSC film was deposited on the YSZ film at T_sub_ = 25 °C with P = 2.7 Pa. DC sputter was used to coat Pt film on the LSC film. The base pressure was ~10 Pa and the applied current was 25 mA. During the deposition of LSC and Pt film, a custom-made STS mask was used to determine cathode area (~3 mm^2^). For current collection at the anode, Pt mesh (52 mesh, Alfa Aesar) was attached to STS by using Ag paste. The cell was mounted on alumina tube with ceramic sealant (AREMCO, 571-p, USA). Finally, for the current collection at cathode side, Pt mesh was physically contacted with vertical load.

*Characterization*

The electrochemical performances of the cell were evaluated at 450, 500, and 550 °C under 97 % H_2_ + 3 % H_2_O (60 cm^3^ min^-1^) as a fuel gas and open air as an oxidant gas. OCV of the cell was monitored using a programmable electrometer (Model 617, Keithley, USA) during heating to 550 °C at 2 °C min^-1^, which was measured using a thermometer (Model 740, Keithley, USA). The temperature of the cell was measured using R-type thermocouple which was positioned ~1 mm above YSZ electrolyte during test. Impedance spectra were measured using an AC impedance analyzer (VSP, Bio Logic Science instruments, France) with a frequency range of 10^-1^ Hz to 10^6^ MHz and AC signal amplitude of 10 mV. At the same time, current-voltage-power characteristics (I-V-P curve) were measured using an electrochemical interface (VSP, Bio Logic Science instruments, France). The phase of LSTN-YSZ on STS was determined using XRD (Model D/MAX-2500/PC, RIGAKU, Japan) employing Cu-K radiation (40 kV, 100 mA); patterns were analyzed using Jade 9 software (Materials Data, Inc., USA). The microstructure of a cross section of the cell was examined using a field emission scanning electron microscope (FE-SEM, Model XL30S FEG, Philips Electron Optics B.V., Netherlands). Pore size was measured by using an image analysis software (ImageJ, National Institute of Health, USA) and surface roughness (root-mean-square) was analyzed by atomic force microscopy (AFM)(Park systems, XE-100, South Korea) in non-contact mode using PPP-NCHR tip (Figure S1 in supplemental info). The electrical conductivity of as-sintered substrate was measured in 97 % H_2_ + 3 % H_2_O using an AC impedance analyzer (Solartron, SI1260, U.K.).
